# Transcriptome characterization of moso bamboo (*Phyllostachys edulis*) seedlings in response to exogenous gibberellin applications

**DOI:** 10.1186/s12870-018-1336-z

**Published:** 2018-06-20

**Authors:** Hangxiao Zhang, Huihui Wang, Qiang Zhu, Yubang Gao, Huiyuan Wang, Liangzhen Zhao, Yongsheng Wang, Feihu Xi, Wenfei Wang, Yanqiu Yang, Chentao Lin, Lianfeng Gu

**Affiliations:** 1 Basic Forestry and Proteomics Research Center, College of Forestry, Fujian Provincial Key Laboratory of Haixia Applied Plant Systems Biology, Fujian Agriculture and Forestry University, Fuzhou, 350002 China; 2 College of Life Science, Fujian Agriculture and Forestry University, Fuzhou, 350002 China; 3 Department of Molecular, Cell & Developmental Biology, University of California, CA90095, Los Angeles, USA

**Keywords:** Gibberellins, PacBio single molecule real time sequencing, Nature antisense transcripts, Alternative splicing, Moso bamboo

## Abstract

**Background:**

Moso bamboo (*Phyllostachys edulis*) is a well-known bamboo species of high economic value in the textile industry due to its rapid growth. Phytohormones, which are master regulators of growth and development, serve as important endogenous signals. However, the mechanisms through which phytohormones regulate growth in moso bamboo remain unknown to date.

**Results:**

Here, we reported that exogenous gibberellins (GA) applications resulted in a significantly increased internode length and lignin condensation. Transcriptome sequencing revealed that photosynthesis-related genes were enriched in the GA-repressed gene class, which was consistent with the decrease in leaf chlorophyll concentrations and the lower rate of photosynthesis following GA treatment. Exogenous GA applications on seedlings are relatively easy to perform, thus we used 4-week-old whole seedlings of bamboo for GA- treatment followed by high throughput sequencing. In this study, we identified 932 *cis*-nature antisense transcripts (*cis*-NATs), and 22,196 alternative splicing (AS) events in total. Among them, 42 *cis*-nature antisense transcripts (*cis*-NATs) and 442 AS events were differentially expressed upon exposure to exogenous GA_3_, suggesting that post-transcriptional regulation might be also involved in the GA_3_ response. Targets of differential expression of *cis*-NATs included genes involved in hormone receptor, photosynthesis and cell wall biogenesis. For example, *LAC4* and its corresponding *cis*-NATs were GA_3_-induced, and may be involved in the accumulation of lignin, thus affecting cell wall composition.

**Conclusions:**

This study provides novel insights illustrating how GA alters post-transcriptional regulation and will shed light on the underlying mechanism of growth modulated by GA in moso bamboo.

**Electronic supplementary material:**

The online version of this article (10.1186/s12870-018-1336-z) contains supplementary material, which is available to authorized users.

## Background

Gibberellins (GA), are one of the most important class of growth-promoting phytohormones, play crucial roles in many aspects of plant growth and development, especially in growth promotion and flower induction [1–3]. GA regulate growth by suppressing a group of DELLA nuclear repressor proteins [1], and display extensive cross-talk with other hormone signaling pathways, including auxin and brassinosteroids (BR) [4–6]. Moso bamboo, native to China and Taiwan, is now grown worldwide for commercial purposes thanks to its very fast growth [7, 8]. Transcriptome analysis in the shoots of moso bamboo revealed hormone-related genes are involved in both growth directions, that is in height and in thickness [9, 10]. A later proteomics study showed seven protein spots involved in hormone biosynthesis during shoot development [11]. The concentration of endogenous GA_3_, one of most common bioactive forms of gibberellic acid [12], displays bimodal variation when the above-ground heights grow from 0.05 m to 12 m in moso bamboo [11, 13, 14]. The extensibility of moso bamboo cell walls is a key factor in moso bamboo’s growth, as they allow grow to great heights due to the two primary components of bamboo walls, cellulose and lignin [15]. Lignin, a main resource for biofuels, functions as a transport vessel to maintain the structural and mechanical integrity of the entire plant [16]. Cellulose fibrils maximize longitudinal elasticity while lignification primarily increases the transversal rigidity of the fibrils [17], which overall provides structural support. Proteins involved in cell wall biosynthesis are reported to be related to rapidly elongating bamboo culms [11]. Although endogenous hormone studies have been conducted in the shoots of moso bamboo [18], the direct link between hormone activity and post-transcriptional regulation remains unknown. In addition to post-transcriptional regulation by alternative splicing (AS), natural antisense transcripts (NATs) also play important roles. NATs take part in post-transcriptional regulation by partial or complete complementation of other transcripts [19–21]. *Cis*-NATs predominantly regulate the same genomic loci with perfect complementarity, which can be categorized into three types: head-to-head, tail-to-tail, and fully overlapping [22]. Genome-wide identification of NATs in mouse, human, rice, and *Arabidopsis thaliana* revealed that natural antisense transcription is a widespread phenomenon [23–29]. In this study, we provide the genome-wide analysis of *cis*-NATs in moso bamboo using PacBio single-molecule real-time sequencing (SMRT) [30]. In combination with RNA-Seq in this study, we revealed differential *cis*-NATs and AS events upon GA induction, which provides novel insight into GA-mediated post-transcriptional regulation in moso bamboo.

## Results

### Exogenous gibberellin applications result in increased internode elongation in moso bamboo

**Fig. 1:**
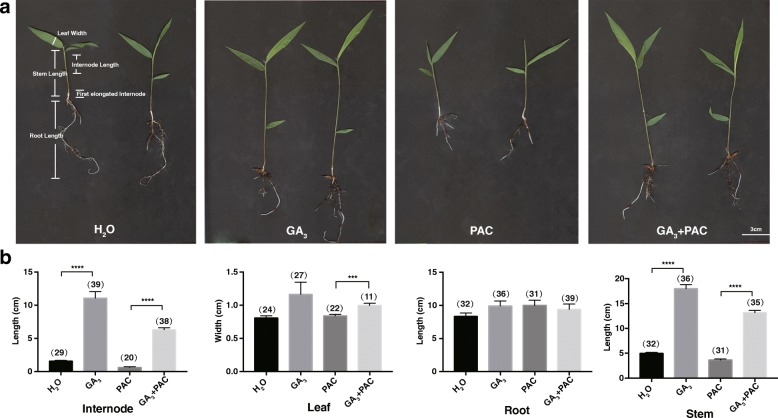
Phenotype of GA-treated bamboo seedlings. **a**: Effects of GA_3_, PAC or GA_3_ + PAC on moso bamboo. Two-week-old bamboo seedlings were treated with H_2_O, GA_3_ (100 μM), PAC (200 μM) every day for two weeks, and phenotypic characteristics were then recorded. Internode length: the length between second and third leaf. Stem length: from the top node to the first node. Root length: the length of primary root. Leaf width: the width of the leaf at the widest point. First elongated internode: the internode between second and third node. White line represents 3 cm in that pixel. **b**: Statistical analysis of the effects of GA_3_, PAC or GA_3_ + PAC on internode length, leaf width, root length and stem length of moso bamboo. Values were Mean ± SEM from replicates, and the bars represent SEM. Student t test was used to evaluate the difference at the 0.05 probability level. Asterisk indicates a significant difference. “***”: *P* < 0.001; “****”: *P* < 0.0001. The number of seedlings is presented in the parentheses on the bars

To determine whether GA is involved in bamboo growth, 2-week-old bamboo seedlings were treated daily with GA_3_ or the paclobutrazol (PAC, a specific inhibitor of GA biosynthesis inhibitor) for two weeks, and the effects on moso bamboo’s growth were then observed (Fig. 1a). Internode and stem lengths significantly increased in moso bamboo treated with exogenous GA_3_ applications. In contrast, PAC significantly retarded stem elongation of moso bamboo as previously reported from many other species [31–33], and exogenous GA_3_ could reverse the inhibition of PAC (Fig. 1b). Leaf width was also sensitive to GA_3_, whereas root length was not altered by GA_3_ applications. The results showed that GA_3_ promoted stem growth of bamboo mainly by affecting internode elongation.

### Transcriptome-scale analysis of GA-responsive genes

**Fig. 2:**
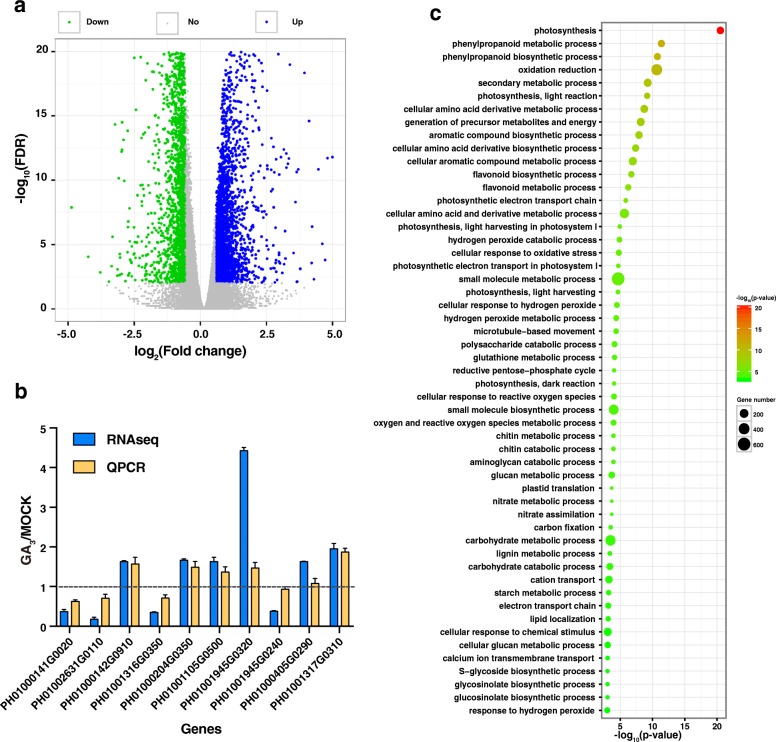
Global analysis of genes expression in response to exogenous gibberellins. **a**: Volcano plot of differentially expressed genes with the cutoff (fold-change > 1.5 and FDR < 0.01). Colors of blue and green represent up- and down-regulated genes, respectively. **b**: Comparison of RNA-seq and qRT-PCR data. RNA-seq data and qRT-PCR quantification of changes of 10 selected differentially expressed genes in GA_3_- and H_2_O-treated seedlings. Data represent the median fold-change value for 3 replicates. The fine dotted line indicates expression levels were equal in GA_3_- and H_2_O-treated seedlings. **c**: Most significant GO of differential expressed genes are presented graphically. The X-axis represents the value of -log_10_(*p*-value). The Y-axis indicates the name of GO term. The size of each point is proportional to the differentially expressed genes associated with the GO terms

To better understand the underlying molecular mechanism, we used RNA-Seq to profile the transcriptomes of GA-treated and non-treated moso bamboo. In total, we obtained 130,988,922 and 153,580,552 reads with three biological repeats from GA-treated and non-treated control libraries, respectively. The mapping rate for each library was above 88%. We obtained 47,927,346 and 56,985,350 uniquely mapped reads for further analysis (Additional file 1: Table S2). By comparative analysis, we identified 5148 significantly differentially expressed genes (DEGs), of which 3025 were GA-induced and 2123 were GA-repressed (Fig. 2a, Additional file 2: Table S3). To verify the results from RNA-Seq, 14 genes were selected for further confirmation by qRT-PCR, and 10 of these genes showed expression profiles consistent with the RNA-Seq results, including 6 GA-induced and 4 GA-repressed genes, indicating that the RNA-Seq data was reliable (Fig. 2b,Additional file 3: Table S1). Gene Ontology (GO) analysis showed that these DEGs are involved in photosynthesis, lignin metabolic process, response to chemical stimulus and others (Fig. 2c).

### GA response and biosynthetic genes in response to exogenous GA

**Fig. 3:**
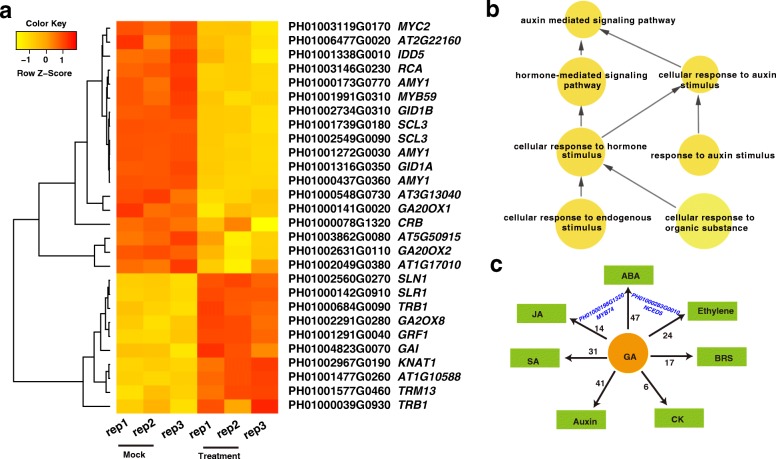
Differential gene expression in the GA signaling pathway and other hormone-related signaling pathways. **a**: Heat map of the GA related genes with significant differential expression upon GA_3_ treatment. **b**: GO enrichment analyses for DEGs. **c**: Crosstalk with other hormone-related signaling pathways. The digit indicates the number of GA-responsive genes involved in the corresponding hormone-related pathway. *MYB74* and *NCED5* were found in two distinct pathways

From GO enrichment analysis, 61 up-regulated DEGs were assigned to the functions of “hormone-mediated signaling pathway” and “cellular response to hormone stimulus” (Additional file 4: Table S4). In total, 28 genes involved in “GA biosynthetic process” and “responding to GA” significantly changed in response to the application of exogenous GA (Fig. 3a). For example, the expression of *GA20OX1,* a GA-biosynthesis gene [34], was reduced upon exogenous GA treatment in moso bamboo. Additionally, the GA receptors *GID1A* and *GID1B* were down-regulated, and the GA repressor genes *SLR1, SLN1,* and *GAI* were up-regulated. Since the concentration of exogenous GA was saturated in our study, the observed repression of GA biosynthesis and signaling genes may be due to the negative feedback regulation of endogenous GA biosynthesis in response to the high concentrations of exogenous GA (Fig. 3a).

### Other hormone-related signaling pathways upon exogenous GA applications

Interestingly, genes associated with other hormone signaling pathways were also significantly overrepresented upon GA_3_ treatment (Fig. 3b, Additional file 4: Table S4). For example, GO terms associated with responses to auxin were also enriched. These results support the idea that the GA signaling pathway crosstalks with the auxin signaling pathway, which is consistent with other previously well-studied plant species [4]. In moso bamboo, 883 hormone-related genes were identified from the search for homologs of the *Arabidopsis* horone database [35]. Among them, 188 were significantly differentially expressed, and they were mainly involved in the eight principal classes of plant hormones: abscisic acid (ABA), auxin, BR, cytokinins (CK), ethylene, GA, jasmonates (JA) and salicylic acid (SA) (Fig. 3c, Additional file 5: Table S5). This study indicates that, at least in moso bamboo, exogenous GA applications can affect other hormone-related pathways, which is consistent with previous studies [36, 37].

### Exogenous GA treatment influences photosynthesis

**Fig. 4:**
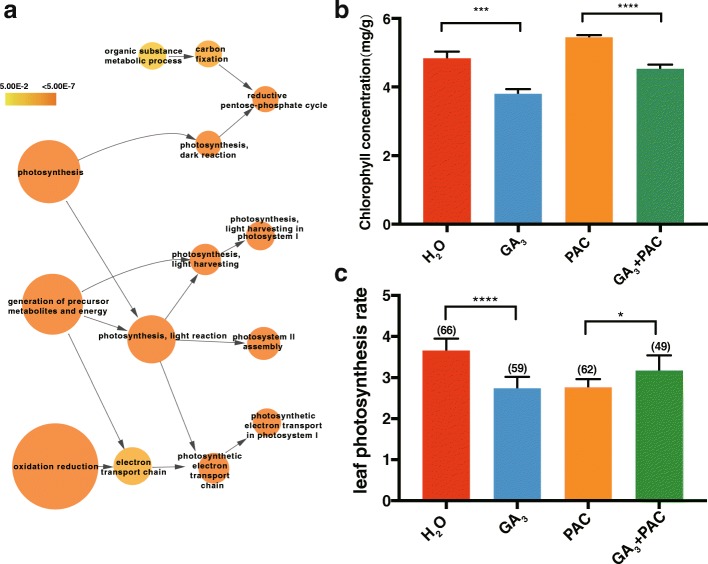
GA_3_ reduced photosynthesis activity. **a**. Photosynthesis associated GO terms are enriched in sets of GA-repressed genes. **b**. Determination of chlorophyll content of H_2_O-, GA_3_, PAC and GA + PAC. **c**. The photosynthesis net rate of four distinct treatments. Asterisk indicates a significant difference.“*”: *P* < 0.05; “***”: *P* < 0.001; “****”: *P* < 0.0001

Among the identified GA-repressed genes, functions related to photosynthesis were most enriched (Fig. 4a, Additional file 4: Table S4), prompting us to hypothesize that GA treatment may affect photosynthesis in GA_3_-treated bamboo. To test this, leaf chlorophyll content was measured in GA_3_, H_2_O, both GA_3_ and PAC, and PAC treated seedlings (Fig. 4b). The results showed that the chlorophyll content was increased in the PAC treated seedlings, while reduced in exogenous GA_3_ treatments, providing additional evidence that GA_3_ can affect photosynthetic capacity.

To further strengthen the connection between GA and photosynthesis, the leaf photosynthetic rate was also measured, and the results showed that exogenous GA_3_ treatment reduced the photosynthetic rate in comparison with H_2_O-treated seedlings (Fig. 4c). PAC reduced the photosynthetic rate, and subsequent exogenous GA_3_ treatment partially reversed the PAC-induced repression. These results suggested that exogenous GA treatment may affect the regulation of photosynthesis to adjust the carbon cycle to the development of seedlings.

### Genes involved in the formation of cell walls

**Fig. 5:**
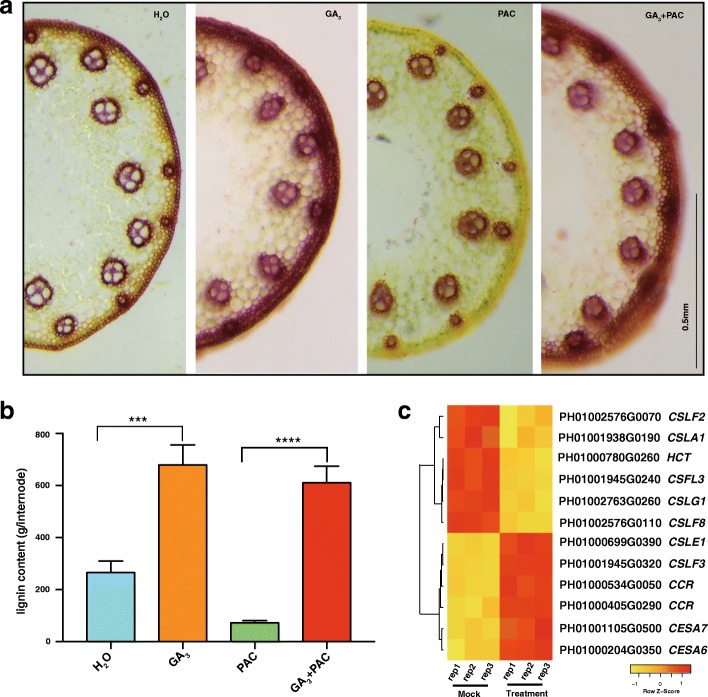
DEGs involved in cell wall structure and lignification. **a**. Transverse sections of first internode of H_2_O, GA_3_, PAC and GA + PAC treatments. **b**. Lignin content in the first internode treated with H_2_O, GA_3_, PAC and GA + PAC. Asterisk indicates a significant difference. “***”: *P* < 0.001; “****”: *P* < 0.0001. **c**. Heatmap of cellulose synthase genes (*Ces A*), cellulose synthase-like genes (*Csl*) and lignin biosynthesis related genes

Based on transverse sections of the first elongated internode of stems, more intense staining was found in the vessels and fibers after the application of bioactive GA_3_, thus indicating increased lignification. In contrast, PAC treatment dramatically reduced lignification (Fig. 5a). In addition, exogenous GA_3_-treatment rescued the effects of PAC. These histological observations were further confirmed by the lignin content determination (Fig. 5b). Together, these results showed that GA treatment significantly increased the lignification of stems (*P* < 0.001), similarly to findings with winter wheat, dwarf pea and tobacco [38–41].

Given that the cell wall architecture is a key determinant for plant growth, we focused on 66 cell-wall-related genes, which are clustered into three groups: cellulose synthase genes (*Ces A*), cellulose synthase-like genes (*Csl*) and lignin biosynthesis related genes [7]. Among them, we identified 12 DEGs. Two members of *CesA* gene family, *CESA6* and *CESA7,* were dramatically up-regulated upon GA treatment (Fig. 5c). *CSLF8, CSLG1, CSLA1* and *CSLF2* were repressed in response to GA applications*,* while *CSLE1* was GA-induced (Fig. 5c). Cinnamoyl-CoA reductase (CCR) catalyzes the first step in the monolignol biosynthetic pathway, and a higher concentration of CCR protein corresponds to increase in lignification [42, 43]. In moso bamboo, GA-induced *CCR* gene suggested that they might take part in the regulation of lignification biogenesis. Considering the significant up-regulation of *CCR*, we propose that the accumulation of lignin after GA_3_ treatment may result from the transcriptional activation of lignin biosynthesis related genes.

### GA affects the AS

**Fig. 6:**
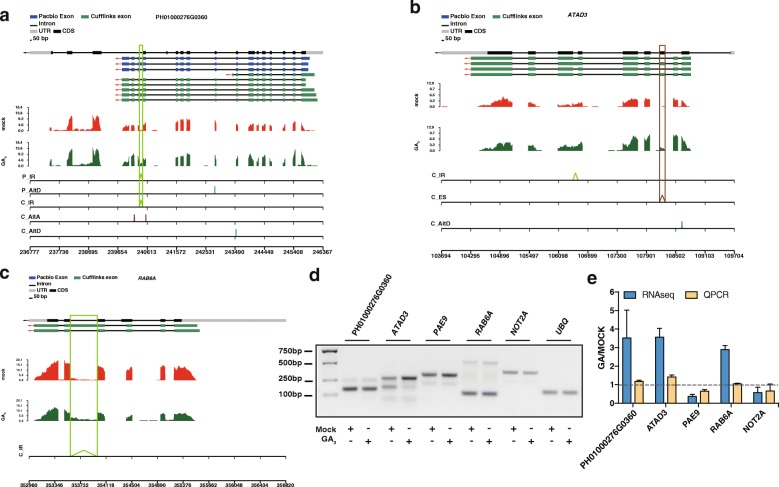
Five genes with AS events were affected by GA_3_. **a**-**c**. Wiggle track for three candidate genes, which have differential isoforms after GA3 treated. Gene structures in blue originated from Pacbio SMRT dataset, while green were generated by Cufflinks assemble of RNA-Seq. “P” indicates the AS events predicted with Pacbio SMRT data, and “C” represented events from RNA-Seq data. **d**. AS events from five genes were validated by RT-PCR gel blot with *UBQ* as the positive control. “+” indicated GA treatment, and “-” represented non-treatment. **e**. Validation of AS events by qRT-PCR

During shoot growth, 60% of genes display alternatively spliced isoforms in moso bamboo [44]. According to cufflink assembler, we identified 233,495 unique coordinates for introns, which represented splice-junction regions. Among these splicing junction sites, we identified 22,196 AS events in seedlings using RNA-Seq. Among them, there were 442 differential AS events classed into four distinct types: 113 intron retention (IR), 120 exon skipping (ES), 135 alternative acceptor (AltA), 74 alternative donor (AltD) (Additional file 6: Table S6). For example, *RCA* (PH01003146G0230), which is involved in the response to light stimulus [45], was found to generate different isoforms by AltD. RT-PCR and qRT-PCR with isoform-specific primers further validated several selected AS events, such as PH01000276G0360*, ATAD3, PAE9, RAB6A* and *NOT2A*, which further suggested that RNA-Seq was reliable (Fig. 6, Additional file 3: Table S1). Longer isoforms of *ATAD3, PAE9* and *RAB6A* were up-regulated, and *NOT2A* was down-regulated upon exogenous GA_3_ treatment (Fig. 6). *ATAD3,* a mitochondrial membrane ATPase*,* is highly conserved from human to plants [46, 47]. *ATAD3* generated two isoforms with an alternative exon skipping, and GA treatment repressed the exon skipping and generated longer isoform (Fig. 6b). The additional exon encoded a potential 39 aa in the coiled-coil region by a homology search in *C.elegans* (UniProt, Q20748). Further experiments are needed to study whether exon skipping of *ATAD3* affects its subcellular localization or function.

In total, 19 genes with GA-responsive splice isoforms, were related to hormone signaling pathway (Additional file 7: Table S7). For example, *PIF1* (IR, PH01001389G0560) is involved in GA signaling pathway, which directly regulates *DELLA* (*GAI* and *RGA*) (Additional file 7: Table S7) [48]. Additionally, *GRF1* (IR, PH01001291G0040) takes part in the gibberellin signaling pathway; it is reported to associate with stem elongation in rice [49] and UV-B repression in maize [50]. In addition, we identified 9 genes encoding splicing factors, generating different isoforms after GA treatment (Additional file 7: Table S7). It is important to note that two splicing factor genes, *RNP_N1* (PH01000290G0450:ES), *RSP40/RSP35* (PH01000347G1190:ES) are also hormone-related [51], and have differentially expressed AS events. Overall, these results strongly suggest that AS accommodates to GA_3_ action in moso bamboo.

### GA affects the NATs

**Fig. 7:**
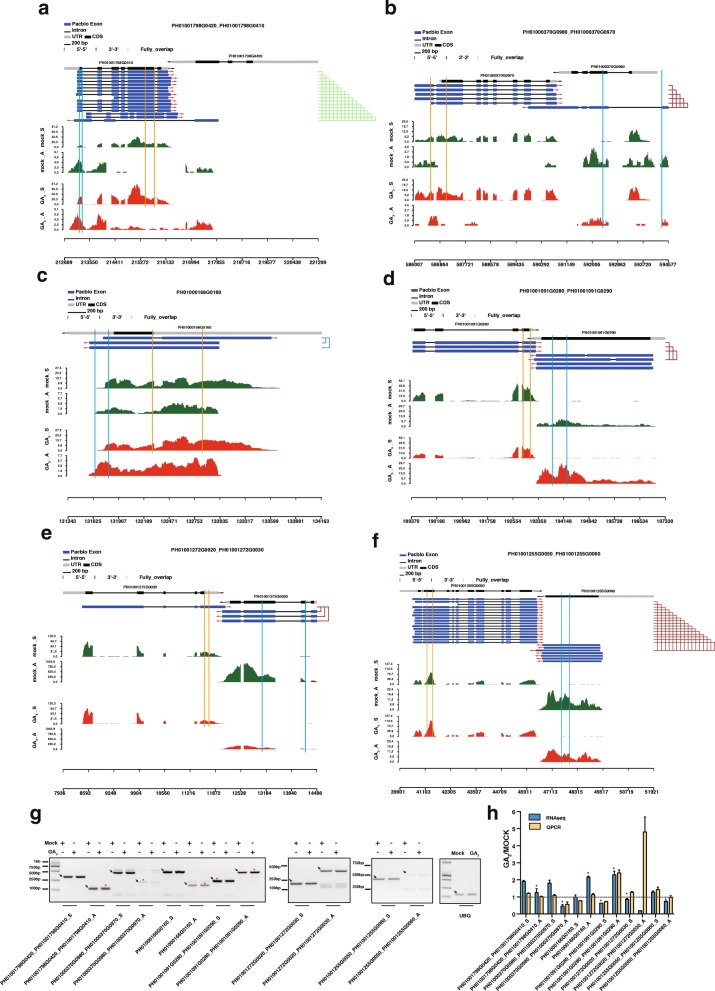
Visualization and validation of *cis*-NATs. **a**-**f**. Wiggle track for six pairs of *cis*-NATs*.* **g**. RT-PCR validation of six pairs of *cis*-NATs as above. The black arrows indicate the target band. The RT-PCR and qRT-PCR validation of these *cis*-NATs. The black asterisk represents the differential cis-NATs, and the fine dotted line indicates the expression levels were equal between GA_3_ and mock treatments. **h**. Valiation of *cis*-NAT using qRT-PCR

*Cis*-NATs regulate transcripts from the same genomic loci with perfect complementarity. Using PacBio full-length transcripts, we revealed genome-wide NATs and identified 932 *cis*-NATs classed into three types: head-to-head (5′-5′), tail-to-tail (3′-3′) and fully overlapping (Additional file 8: Table S8). Genome-wide analyses showed that most NATs are generated from long noncoding RNA (lncRNA) in both mammals [52] and plants [53]. Among the 932 *cis*-NATs in moso bamboo, there were 492 pairs of *cis*-NATs including long noncoding RNAs, which represented long noncoding natural antisense transcripts (lncNATs). In total, there were 42 differentially expressed *cis*-NATs (Additional file 9: Table S9). We selected 6 pairs of *cis*-NATs for further validation by RT-PCR, and all of them could be amplified with the expected band (Fig. 7), demonstrating the advantage of the identification of NATs using PacBio full-length transcripts. Among them, the changes of 4 pairs were apparently identical to in silico analysis, while the other two were not obvious by RT-PCR. It is possible that this discrepancy is due to the limited sensitivity of agarose gel electrophoresis, so real-time PCR was carried out. qRT-PCR results showed expression profiles consistent with RNA-Seq results except for one pair of *cis*-NATs (Fig. 7), which suggested the ability of GA to regulate NATs.

**Fig. 8:**
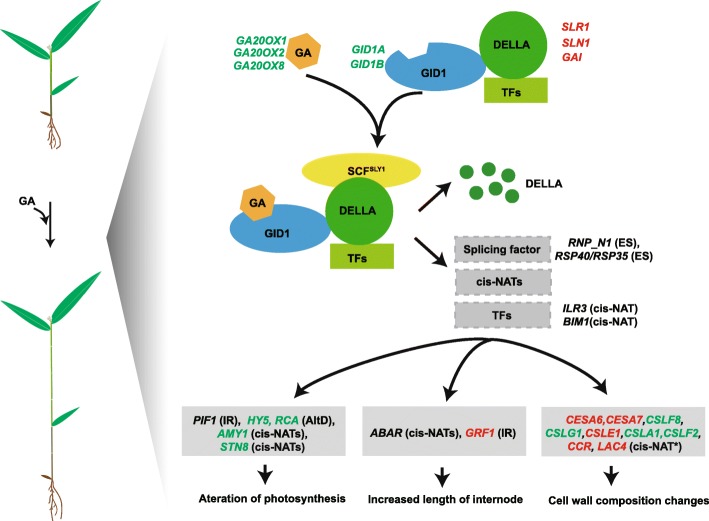
A model for the possible mechanism of exogenous GA regulation of growth in moso bamboo at the transcriptional and post-transcriptional levels. Genes marked in red indicates that these genes were GA-induced, and likewise, green ones indicates that the genes were GA-repressed. Content in brackets included the predicted post-transcriptional regulation modes. “*” represented genes with experimental evidence

In total, there are 3 and 21 *cis*-NATs targeted genes involved in “hormone metabolic process” and “response to endogenous stimulus”, respectively. Four genes are part of “cell wall biogenesis”, and 43 genes were assigned to the function of “oxidation reduction” (Additional file 10: Table S10). In this study, we revealed that *ABAR* (PH01000056G0440) and *AMY1* (PH01001272G0030) in moso bamboo were regulated by *cis*-NAT PH01000056G0430 and PH01001272G0020, respectively (Fig. 8), and it has been reported that *ABAR* and *AMY1* are associated with hormone receptor [54] and gibberellin [55]. Our data showed that the kinase gene *STN8* (PH01003235G0120) is involved in phosphorylation of photosystem II core protein, and could be targeted by *cis*-NATs, which are located in the intron of *STN8*. The transcript abundance of *STN8* was dramatically down-regulated upon GA-induced growth (Fig. 8). These results indicated that *cis*-NATs may participate in reducing photosynthetic capacity in response to GA_3_ treatment. *LAC4* is involved in constitutive lignification of stems together with *LAC17* [56]. It is important to point out that in bamboo, GA-induced *LAC4* (PH01001798G0410) and its corresponding cis-NATs are both GA-inducible and affect cell wall biogenesis (Fig. 7a). The data shows that *cis*-NATs may be another way to regulate moso bamboo’s growth upon GA treatment.

### Transcript and post-transcriptional regulation of TFs

A previous study showed that GA signaling regulates the activity and protein stability for TFs of PIF3 and HY5 [57]. *HY5* acts as a convergence point of light and multiple hormone signaling pathways [58–60]. According to the homologs search, we identified 678 TFs. Among them, 132 TFs are DEGs, including *HY5* (PH01000075G0960), a bZIP transcription factor (Fig. 8 and Additional file 11: Table S11). TFs affect AS by influencing the transcription elongation rate of RNA polymerase II [61]. Reciprocally, AS also affects the splicing of TFs, and 13 out of 678 TFs have differential AS events (Additional file 12: Table S12). For example, *PIF1* (PH01001389G0560), an important hub gene in both GA and light signaling pathway [48], and it is regulated by IR (Fig. 8). In addition, *ILR3* (PH01000773G0410) and *BIM1* (PH01004969G0010) have complementary loci to *cis*-NATs, which imply that TFs may also be under the regulation of NATs (Fig. 8), though more evidence is required to elucidate this.

## Discussion

### Gibberellin affects photosynthesis

Earlier published studies revealed a contradictory relationship between GA and photosynthesis [62, 63]. Some studies reported a positive effect of GA on photosynthesis [64, 65], while others showed a decreased rate of photosynthesis after exogenous GA applications [66], and one study reported no difference [67]. This study uncovered that exogenous GA_3_ significantly reduced chlorophyll content, in agreement with the decreased photosynthetic rate, which was similar to the findings with the the slow-growing inbred lines of *Plantago major* L. [66]. A study in *Populus* showed that the same concentration of GA_3_ plays a positive role in regulating photosynthesis [68]. The difference may be due to the different conditions of the treatment or different developmental stages. This study used GA_3_ to treat two-week-old seedlings daily for two weeks. However, the study in *Populus* used one year old plants with weekly GA treatments for one month [68]. At present, the effect of PAC on photosynthetic efficiency is also not clear. It is reported that PAC has different effects, including no effect on photosynthetic rate in ‘delicious’ apples [69], negative effect in wheat and sweet orange [70, 71], and positive effect in radish and *Catharanthus roseus* [32, 72]. In this study, PAC reduced the photosynthetic net rate slightly, which may be due to the PAC concentration being slightly high and resulting in a phytotoxia reaction. A series of treatments of different concentration of GA with a time-course should be carried out to elucidate the exact relationship between GA, PCA and photosynthesis.

### Gibberellin affects lignification

In this study, we found that GA_3_ treatment increased lignification, which was consistent with previous studies in other plant species [38–41]. At present, we only investigated the short-term effects of GA_3_ treatments on lignification. In the future, it will be interesting to observe the long-term effects of GA_3_ on the biosynthetic pathway of lignification, which has been reported to differ from the short-term effects [40].

The higher expression of *LAC4* results in accumulation of lignin, which is also observed in *C.sinensis* [73]. As mentioned above, *LAC4* is a GA-induced lignification related gene, which was found to be regulated by cis-NATs in moso bamboo. It is also reported to be regulated by transcription factors, for example MYB58 and MYB63 [74]. In moso bamboo, *MYB58* (PH01000386G0660) was GA-repressed and *MYB63* (PH01000030G0050) was GA induced. In addition, microRNAs may also be involved in regulating the expression of *LAC4.* For example, miR397 is predicted to target the fifth exon of *LAC4* and act as a negative regulator of laccases transcripts [75]. Recent studies show that miR397a and miR397b regulate lignin content by regulating the laccase genes, for example *LAC4* [73, 76]. Thus, we propose that up-regulation of *LAC4* in moso bamboo results from the action of multiple players, such as TFs, cis-NATs and even microRNA, suggesting the existence of diversity in the regulation network of lignin upon GA treatment.

### Gibberellin affects AS

Though previous studies showed that *HAB1* generates two spliced isoforms, *HAB1.1* and *HAB1.2*, which function as opposite regulators in ABA sensitivity [77], there is a lack of genome-wide analysis of AS upon hormone treatment. The comparison of AS changes between mock and GA treatment samples allowed us to identify several hundreds of differential AS events. In this study, we identified 442 differential AS events. Experimental validation showed that qRT-PCR with isoform-specific primers was more sensitive than RT-PCR to quantify the expression of the isoforms. In this study, the longer isoform of *ATAD3* was GA induced (Fig. 6b). Searches of public database revealed that *ATAD3* is relatively conserved in *Arabidopsis*, *Oryza sativa* and *Brachypodium distachyon*, but is not yet well studied in other plants. Further bioinformatics analysis showed that the skipped exon encoded 39 aa within the coiled-coil region, which suggested that exon skipping functions in the interaction with partners [46]. More experiments are needed to study whether the GA-affected exon skipping of *ATAD3* affects its subcellular localization or functions. *ATAD3* may be a good case study to investigate the links between the different isoforms and distinct function, and will provide a better understanding of how GA-induced AS affects the growth and development of moso bamboo.

### Gibberellin affects NATs

In this study, we took advantage of the full-length transcripts from PacBio sequencing to profile 932 *cis*-NATs, which avoids likely error-containing transcriptome assembly. GA regulates growth throughout the plant life cycle. Thus, the number of *cis*-NATs is underestimated and more differential *cis*-NATs will be revealed using more time points or tissues. In this study we investigated the expression of 932 *cis*-NATs using strand-specific library, and revealed part differential *cis*-NATs with experimental validation using both RT-PCR and qRT-PCR. A well-defined natural antisense gene pair is *P5CDH* and *SRO5*, which overlaps with each other by the tail-to-tail model and generates endogenous nat-siRNA related with salt stress [78]. Our data revealed that many GA-responsive genes in moso bamboo, such as *ABAR*, *AMY1, STN8* and *LAC4,* may be regulated by *cis*-NAT. However, there is no adequate experimental evidence to show whether they generate differential nat-siRNAs. In the future, the nat-siRNA from the complementary regions of overlapping transcripts can be investigated using small RNA sequencing methods, which is optimized for the identification of the potential mechanism of *cis*-NATs. Except for nat-siRNA, the expression of antisense RNA induces the silencing and methylation of corresponding genes [79]*.* It will be interesting to reveal the role of NAT-mediated DNA methylation in response to GA in the future.

FLC (FLOWERING LOCUS C) acts as a repressor of flowering, which is a well-characterized example for lncNATs. The antisense transcripts of *FLC* are induced by cold and the non-coding transcripts act to silence *FLC* transcription transiently [80]. In this study, we found that more than 50% pairs of *cis*-NATs included lncNATs. However, the mechanism of lncNATs remains unknown in moso bamboo. At present, a method of regeneration and transformation is still lacking in moso bamboo. Overexpressing antisense transcripts in protoplasts might reveal stage-dependent regulation of lncNATs in the corresponding sense transcription.

## Conclusions

In order to reveal how GA regulates growth in moso bamboo, we applied exogenous gibberellins (GA_3_) on moso bamboo seedlings, and observed that it could significantly increase internode length. Determination of chlorophyll content and photosynthetic rate revealed that GA had a negative effect on the photosynthetic activity in moso bamboo. In addition, lignin visualization with free-hand sections showed the increased accumulation of lignin in response to GA treatment, implying a functional role of GA in bamboo growth by affecting cell wall composition. Taking full advantage of the PacBio single-molecule real-time sequencing (SMRT), we provided the first genome-wide analysis of *cis*-NATs in moso bamboo. In combination with strand-specific RNA-Seq, we revealed differential *cis*-NATs and AS events upon GA induction, which further suggested that post-transcriptional regulation is also involved in the GA response in moso bamboo. This study provides novel insights into GA-mediated post-transcriptional regulation on growth and development in moso bamboo.

## Methods

### Plant materials

For RNA sequencing, three biological replicates of 4-week-old whole seedlings grown under long-day conditions (16 h light/8 h dark) were collected from MS (Murashige and Skoog) medium after being treated with H_2_O or GA_3_ (100 μM) for 4 h. The plant materials were snap frozen in liquid nitrogen and stored at − 80 °C for total RNA extraction and library construction. Total RNA was isolated with the RNAprep Pure Plant Kit (Tiangen, Cat. #DP441, China) following the manufacturer’s protocol. RNA quality was assessed using an agarose gel and NanoDrop 2000c UV-Vis Spectrophotometer (ThermoFisher, USA). The integrity of RNA samples was further evaluated using an Agilent 2100 Bioanalyzer (Agilent Technologies, Santa Clara, CA, USA). Two-week-old bamboo seedlings were sprayed with H_2_O or GA_3_ (100 μM), PAC (200 μM) seven times a week for two weeks, and their phenotypic characteristics were recorded. Chlorophyll content, photosynthetic rate and lignin content were analyzed with the above treatment material.

### Determination of chlorophyll and photosynthetic rate

To determine the chlorophyll content, ~ 0.1 g fresh leaf material was cut into pieces and added to 10 ml of an ethanol, acetone and distilled water (4.5:4.5:1, *v*/v/v) mixture to extract chlorophyll. Then the 15 ml centrifugation tube was wrapped in aluminium foil and placed in the dark for three days, until the sample turned white. Finally, 3 ml of each chlorophyll solution were taken from the tube and added to a 10 mm cuvette to measure optical density at 645 nm and 663 nm with Thermo Fisher Multiskan™ FC microplate photometer. The experiments were performed with at least 6 replicates and chlorophyll content was then calculated using Arnon’s equations [81]:

$$ \left(20.2\times {A}_{645}+8.02\times {A}_{663}\right)\times \left(\mathrm{V}/\mathrm{W}\right) $$

Net photosynthetic rate of bamboo seedlings was calculated with the LI-6400XT Portable Photosynthesis System (LI-COR Corporate, USA) under normal growing conditions. More than 30 first fully expanded leaves of distinct seedlings were used for each treatment. Each leaf was measured with five repeats to obtain an average value. These leaves were marked sequentially and scanned with a Microtek ScanMaker i800 plus system (WSeen, Hangzhou, China). ImageJ was used to calculate the area of leaves accurately. Finally, the photosynthetic rate was calculated using the formula (CO_2_R-CO_2_S*(1000-H_2_OR)/(1000-H_2_OS))*fda, where CO_2_R and CO_2_S was the CO_2_ concentration from reference and sample cell, respectively. H_2_OR and H_2_OS was H_2_O concentration from reference and sample cell, respectively. Fda was referred Flow/Area of leaf.

### Lignin visualization with hand-cut sections and lignin content measurement

For lignin visualization, free-hand sections of 4-week-old seedlings’ first internode were obtained. Then above material was stained with phloroglucinol-HCl (1% (weight/volume) phloroglucinol in 6 mol/l HCl) for 5 min and then observed under a light microscope.

Lignin of the first elongated internode of stems was quantified by modifying the acetyl bromide spectrophotometric method [82]. Samples were ground into powder, 95% ethanol was added immediately and centrifuged for 5 min at 2600 g. The above material was then washed twice with 95% ethanol, after which the pellet was rinsed twice with ethanol-hexane (1:2, v:v). After air drying the samples, the pellets were dissolved with 25% bromine acetyl acetic solution, and then incubated at 70 °C for 30 min. The staining reaction was stopped with 0.18 mL NaOH, 0.02 mL 7.5 mol/L hydroxylamine hydrochloride, and glacial acetic acid was added to reach a final volume of 3 ml. The supernatant was collected after centrifugation at 1000 g for 7 min, and the lignin content was estimated reading the absorbance values at 280 nm.

### Library construction for RNA sequencing

RNA-Seq libraries for H_2_O and GA_3_ treated samples were constructed by the dUTP method according to the manufacturer’s instructions (Illumina, San Diego, CA, USA), as described in our previous study [30]. Paired-end reads (read length = 125 bp*2) were sequenced with Illumina HiSeq™ 2500 (Illumina, San Diego, CA, USA). All the RNA-Seq raw data has been uploaded to NCBI under accession number GSE104596.

### Differential expression analysis

TopHat2 was selected as the mapping tool to map these reads to the moso bamboo reference genome [7, 83] with anchor length more than 8-nt for spliced alignments. Only uniquely mapped reads were retained for subsequent analysis. The bigwig files have been uploaded to our website: http://www.forestrylab.org/pub/GA. The normalized expression levels for gene models were measured as fragments per kilobase of transcript per million mapped reads (FPKM) [84]. False discovery rates (FDR) were calculated using edgeR package [85]. Differential gene expression was identified using fold change > 1.5 and FDR < 0.01.

### Identification of differential AS events

The genome sequence of moso bamboo is available. Thus the identification of alternative splicing is similar to the reference-based method [86, 87]. All 125 bp mapping RNA-Seq paired-end reads were assembled using Cufflinks v2.1.1 [88] with the following option: -F 0.05 -A 0.01 -I 100000 --min-intron-length 30 to generate all potential transcripts including all the exons in this study. Then all above isoforms were combined into one annotation of transcripts in GTF format using the Cuffmerge utility within the Cufflinks v2.1.1 package with the following option: --min-isoform-fraction 0.01. The above assembled isoforms and the mapping reads from for H_2_O and GA_3_ treated samples were loaded into rMATS.3.2.2 [89] to identify differential AS events using the following parameters: -t paired -len 125 -a 8 -c 0.0001 -analysis U. rMATs output includes all the AS events, including intron retention, exon skipping, alternative acceptor, and alternative donor. In total, AS events with *P* < 0.05 were regarded as the differential AS events.

### GO enrichment analysis

Blast2GO optimizes BLAST algorithm to identify homologous sequences and then assigns uncharacterized novel sequences in non-model species with functional annotation, which is represented via the GO vocabulary [90]. In this study, GO terms of each gene in moso bamboo were assigned using BLAST2GO with default option [90], and the enrichment of GO terms was assessed with the BiNGO plugin [39] for Cytoscape [54], using hypergeometric test for statistical analysis. For *P*-value correction, we selected the FDR correction method. GO terms with corrected *P* ≤ 0.05 were considered significantly over-represented and shown as colored nodes in the enrichment network.

### Identification of NATs using PacBio SMRT

PacBio SMRT dataset from our previous study [30] was used for the identification of NATs by our reference-based method [91]. Firstly, the full-length transcripts from PacBio SMRT sequencing with overlapping coordinates from the reference genome were clustered together into transcripts units. Then three types of NATs (head-to-head, tail-to-tail, and fully overlapping) were identified by pair-wise comparison of oppositely oriented full-length transcripts according to overlapping coordinates. Read counts from each NATs were calculated using strand-specific RNA-Seq libraries. The edgeR [85] was used to calculate FDR. Differential NATs were identified using FDR less than 0.01. The lncNATs was predicted using Coding-Non-Coding Index (CNCI) with default option [92].

### Searching for hormone-related and transcript factors

The InParanoid algorithm uses NCBI-Blast as the homology detection tool to calculate pairwise similarity scores [93]. Thus, we searched the homologs in *Arabidopsis* to acquire all the homologous genes in moso bamboo by InParanoid (version 3.0) using the default options [93]. In total, we obtained 1,4139 pairwise homologs. All the hormone-related genes and transcript factors (TFs) in *Arabidopsis* were downloaded from http://ahd.cbi.pku.edu.cn [35] and Steve A. Kay’s study, respectively [94].

### Experimental validation using quantitative real-time PCR

Primers were designed using premier 5 (Premier Biosoft International, Palo Alto, CA, USA) and synthesized at BGI-Tech (Shenzhen, China). All the information for each primer pair can be found in the Additional file 3: Table S1. RNA-Seq results, including differential expression and NATs were validated by qPCR with SYBR® Premix Ex Taq ™ II (TaKaRa, Cat. # RR820A, Japan) on Applied Biosystems QuantStudio™ 6 Flex Real-Time PCR System (ThermoFisher, USA). For each experiment, *NTB* or *UBQ* was analyzed in triplicates as the positive control [95].

### Experimental validation by semi-quantitative RT-PCR

1 μg RNA was used for cDNA synthesis with PrimeScript™ RT reagent Kit with gDNA Eraser (TaKaRa, Cat#RR047A), and PCR was conducted with Premix Taq (TaKaRa Taq Version 2.0 plus dye,Cat#RR901A) in a total 30 μl reaction volume with *UBQ* as the positive control. All the products from AS and NATs were validated with 1% agarose gel electrophoresis.

## Additional files

Additional file 1: **Table S2.** Summary of RNA-Seq mapping. (XLSX 30 kb)

Additional file 2: **Table S3.** List of significant differentially expressed genes. (XLSX 1396 kb)

Additional file 3: **Table S1.** Primer list used in this study. (XLSX 20 kb)

Additional file 4: **Table S4.** GO analysis of DEGs. (XLSX 93 kb)

Additional file 5: **Table S5**. List of 188 significantly altered DEGs engaged in eight principal classes of plant hormones. (XLSX 38 kb)

Additional file 6: **Table S6.** List of differentially AS events including IR, ES, AltA and AltD. (XLSX 207 kb)

Additional file 7: **Table S7**. List of hormone-related and splicing factor genes, which have different isoforms after GA was applied. (XLSX 31 kb)

Additional file 8: **Table S8.** List of *cis*-NATs in moso bamboo, which were classified into head-to-head (5′-5′), tail-to-tail (3′-3′) and fully overlapping. (XLSX 156 kb)

Additional file 9: **Table S9.** List of 42 differentially expressed *cis*-NATs upon GA treatment. (XLSX 14 kb)

Additional file 10: **Table S10.** GO analysis of 42 differentially expressed *cis*-NATs. (XLSX 9 kb)

Additional file 11: **Table S11.** List of 132 differentially expressed TFs. (XLSX 82 kb)

Additional file 12: **Table S12.** List of TFs associated with AS and *cis*-NATs. (XLSX 31 kb)

## Abbreviations

ABA: Abscisic acid
AltA: Alternative acceptor
AltD: Alternative donor
AS: Alternative splicing
CCR: Cinnamoyl-CoA reductase
Ces A: Cellulose synthase genes
*cis*-NATs: *cis*-nature antisense transcripts
CK: Cytokinins
CNCI: Coding-Non-Coding Index
Csl: Cellulose synthase-like genes
DEGs: Differentially expressed genes
ES: Exon skipping
FDR: False discovery rate
FLC: FLOWERING LOCUS C
FPKM: Fragments per kilobase of transcript per million mapped reads
GA: Gibberellins
GO: Gene ontology
IR: Intron retention
JA: Jasmonates
lncNATs: Long noncoding natural antisense transcripts
nat-siRNA: *cis*-NAT-derived siRNAs
SA: Salicylic acid
SMRT: Single-molecule real-time sequencing
TFs: Transcript factors

## References

[1] Claeys H, De Bodt S, Inze D (2014). Gibberellins and DELLAs: central nodes in growth regulatory networks. Trends Plant Sci.

[2] Yu H, Ito T, Zhao Y, Peng J, Kumar P, Meyerowitz EM (2004). Floral homeotic genes are targets of gibberellin signaling in flower development. Proc Natl Acad Sci U S A.

[3] T-p S (2010). Gibberellin signal transduction in stem elongation & leaf growth. Plant hormones.

[4] Nemhauser JL, Hong F, Chory J (2006). Different plant hormones regulate similar processes through largely nonoverlapping transcriptional responses. Cell.

[5] Chaiwanon J, Wang W, Zhu JY, Oh E, Wang ZY (2016). Information integration and communication in plant growth regulation. Cell.

[6] Wang W, Bai MY, Wang ZY (2014). The brassinosteroid signaling network-a paradigm of signal integration. Curr Opin Plant Biol.

[7] Peng Z, Lu Y, Li L, Zhao Q, Feng Q, Gao Z, Lu H, Hu T, Yao N, Liu K (2013). The draft genome of the fast-growing non-timber forest species moso bamboo (Phyllostachys heterocycla). Nat Genet.

[8] Magel E, Kruse S, Lütje G, Liese W (2005). Soluble carbohydrates and acid invertases involved in the rapid growth of developing culms in Sasa palmata (bean) Camus. Bamboo Sci Culture.

[9] Wei Q, Jiao C, Guo L, Ding Y, Cao J, Feng J, Dong X, Mao L, Sun H, Yu F (2017). Exploring key cellular processes and candidate genes regulating the primary thickening growth of Moso underground shoots. New Phytol.

[10] Peng Z, Zhang C, Zhang Y, Hu T, Mu S, Li X, Gao J (2013). Transcriptome sequencing and analysis of the fast growing shoots of moso bamboo (Phyllostachys edulis). PLoS One.

[11] Cui K, He CY, Zhang JG, Duan AG, Zeng YF (2012). Temporal and spatial profiling of internode elongation-associated protein expression in rapidly growing culms of bamboo. J Proteome Res.

[12] Hedden P, Thomas SG (2012). Gibberellin biosynthesis and its regulation. Biochem J.

[13] He CY, Cui K, Zhang JG, Duan AG, Zeng YF (2013). Next-generation sequencing-based mRNA and microRNA expression profiling analysis revealed pathways involved in the rapid growth of developing culms in Moso bamboo. BMC Plant Biol.

[14] Wang HY, Cui K, He CY, Zeng YF, Liao SX, Zhang JG (2015). Endogenous hormonal equilibrium linked to bamboo culm development. Genet Mol Res.

[15] Gritsch CS, Kleist G, Murphy RJ (2004). Developmental changes in cell wall structure of phloem fibres of the bamboo Dendrocalamus asper. Ann Bot.

[16] Rogers LA, Campbell MM (2004). The genetic control of lignin deposition during plant growth and development. New Phytol.

[17] Wang X, Ren H, Zhang B, Fei B, Burgert I (2012). Cell wall structure and formation of maturing fibres of moso bamboo (Phyllostachys pubescens) increase buckling resistance. J R Soc Interface.

[18] Gamuyao R, Nagai K, Ayano M, Mori Y, Minami A, Kojima M, Suzuki T, Sakakibara H, Higashiyama T, Ashikari M, et al. Hormone distribution and transcriptome profiles in bamboo shoots provide insights on bamboo stem emergence and growth. Plant Cell Physiol. 2017; 10.1093/pcp/pcx023 28204696

[19] Wight M, Werner A (2013). The functions of natural antisense transcripts. Essays Biochem.

[20] Rosikiewicz W, Makalowska I (2016). Biological functions of natural antisense transcripts. Acta Biochim Pol.

[21] Wang H, Chua N-H, Wang X-J. Prediction of trans-antisense transcripts in Arabidopsis thaliana. Genome Biol. 2006;7(10):R92. 10.1186/gb-2006-7-10-r92 PMC1794575 17040561

[22] Lapidot M, Pilpel Y (2006). Genome-wide natural antisense transcription: coupling its regulation to its different regulatory mechanisms. EMBO Rep.

[23] Wang X-J, Gaasterland T, Chua N-H (2005). Genome-wide prediction and identification of cis-natural antisense transcripts in Arabidopsis thaliana. Genome Biol.

[24] Li YY, Qin L, Guo ZM, Liu L, Xu H, Hao P, Su J, Shi Y, He WZ, Li YX (2006). In silico discovery of human natural antisense transcripts. BMC Bioinformatics.

[25] Yelin R, Dahary D, Sorek R, Levanon EY, Goldstein O, Shoshan A, Diber A, Biton S, Tamir Y, Khosravi R (2003). Widespread occurrence of antisense transcription in the human genome. Nat Biotechnol.

[26] Zhou X, Sunkar R, Jin H, Zhu JK, Zhang W (2009). Genome-wide identification and analysis of small RNAs originated from natural antisense transcripts in Oryza sativa. Genome Res.

[27] Ansaldi R, Chaboud A, Dumas C (2000). Multiple S gene family members including natural antisense transcripts are differentially expressed during development of maize flowers. J Biol Chem.

[28] Kiyosawa H, Mise N, Iwase S, Hayashizaki Y, Abe K (2005). Disclosing hidden transcripts: mouse natural sense-antisense transcripts tend to be poly(a) negative and nuclear localized. Genome Res.

[29] Osato N, Yamada H, Satoh K, Ooka H, Yamamoto M, Suzuki K, Kawai J, Carninci P, Ohtomo Y, Murakami K (2003). Antisense transcripts with rice full-length cDNAs. Genome Biol.

[30] Wang T, Wang H, Cai D, Gao Y, Zhang H, Wang Y, Lin C, Ma L, Gu L (2017). Comprehensive profiling of rhizome-associated alternative splicing and alternative polyadenylation in moso bamboo (Phyllostachys edulis). Plant J.

[31] Sankar B, Jaleel CA, Manivannan P, Kishorekumar A, Somasundaram R, Panneerselvam R (2007). Effect of paclobutrazol on water stress amelioration through antioxidants and free radical scavenging enzymes in Arachis hypogaea L. Colloid Surface B.

[32] Jabir BMO, Kinuthia KB, Faroug MA, Awad FN, Muleke EM, Ahmadzai Z, Liu LW (2017). Effects of gibberellin and gibberellin biosynthesis inhibitor (Paclobutrazol) applications on radish (Raphanus sativus) taproot expansion and the presence of authentic hormones. Int J Agric Biol.

[33] Hajihashemi S, Geuns JMC (2017). Steviol glycosides correlation to genes transcription revealed in gibberellin and paclobutrazol-treated Stevia rebaudiana. J Plant Biochem Biot.

[34] Rieu I, Ruiz-Rivero O, Fernandez-Garcia N, Griffiths J, Powers SJ, Gong F, Linhartova T, Eriksson S, Nilsson O, Thomas SG (2008). The gibberellin biosynthetic genes AtGA20ox1 and AtGA20ox2 act, partially redundantly, to promote growth and development throughout the Arabidopsis life cycle. Plant J.

[35] Jiang Z, Liu X, Peng Z, Wan Y, Ji Y, He W, Wan W, Luo J, Guo H (2011). AHD2.0: an update version of Arabidopsis hormone database for plant systematic studies. Nucleic Acids Res.

[36] Wang GL, Que F, Xu ZS, Wang F, Xiong AS (2015). Exogenous gibberellin altered morphology, anatomic and transcriptional regulatory networks of hormones in carrot root and shoot. BMC Plant Biol.

[37] Zentella R, Zhang ZL, Park M, Thomas SG, Endo A, Murase K, Fleet CM, Jikumaru Y, Nambara E, Kamiya Y (2007). Global analysis of della direct targets in early gibberellin signaling in Arabidopsis. Plant Cell.

[38] Peng DL, Chen XG, Yin YP, Lu KL, Yang WB, Tang YH, Wang ZL (2014). Lodging resistance of winter wheat (Triticum aestivum L.): lignin accumulation and its related enzymes activities due to the application of paclobutrazol or gibberellin acid. Field Crop Res.

[39] Cheng CK, Marsh HV (1968). Gibberellic acid-promoted lignification and phenylalanine ammonia-lyase activity in a dwarf pea (Pisum sativum). Plant Physiol.

[40] Biemelt S, Tschiersch H, Sonnewald U (2004). Impact of altered gibberellin metabolism on biomass accumulation, lignin biosynthesis, and photosynthesis in transgenic tobacco plants. Plant Physiol.

[41] Townsley BT, Sinha NR, Kang J (2013). KNOX1 genes regulate lignin deposition and composition in monocots and dicots. Front Plant Sci.

[42] Srivastava S, Vishwakarma RK, Arafat YA, Gupta SK, Khan BM (2015). Abiotic stress induces change in Cinnamoyl CoA reductase (CCR) protein abundance and lignin deposition in developing seedlings of Leucaena leucocephala. Physiol Mol Biol Plants.

[43] Giordano A, Liu Z, Panter SN, Dimech AM, Shang Y, Wijesinghe H, Fulgueras K, Ran Y, Mouradov A, Rochfort S (2014). Reduced lignin content and altered lignin composition in the warm season forage grass Paspalum dilatatum by down-regulation of a Cinnamoyl CoA reductase gene. Transgenic Res.

[44] Li L, Hu T, Li X, Mu S, Cheng Z, Ge W, Gao J (2016). Genome-wide analysis of shoot growth-associated alternative splicing in moso bamboo. Mol Gen Genomics.

[45] Carmo-Silva AE, Salvucci ME (2013). The regulatory properties of rubisco Activase differ among species and affect photosynthetic induction during light transitions. Plant Physiol.

[46] Li S, Rousseau D (2012). ATAD3, a vital membrane bound mitochondrial ATPase involved in tumor progression. J Bioenerg Biomembr.

[47] Fan J, Papadopoulos V (2013). Evolutionary origin of the mitochondrial cholesterol transport machinery reveals a universal mechanism of steroid hormone biosynthesis in animals. PLoS One.

[48] Leivar P, Quail PH (2011). PIFs: pivotal components in a cellular signaling hub. Trends Plant Sci.

[49] van der Knaap E, Kim JH, Kende H (2000). A novel gibberellin-induced gene from rice and its potential regulatory role in stem growth. Plant Physiol.

[50] Fina J, Casadevall R, AbdElgawad H, Prinsen E, Markakis MN, Beemster GTS, Casati P (2017). UV-B inhibits leaf growth through changes in growth regulating factors and gibberellin levels. Plant Physiol.

[51] Li H, Wong WS, Zhu L, Guo HW, Ecker J, Li N (2009). Phosphoproteomic analysis of ethylene-regulated protein phosphorylation in etiolated seedlings of Arabidopsis mutant ein2 using two-dimensional separations coupled with a hybrid quadrupole time-of-flight mass spectrometer. Proteomics.

[52] Katayama S, Tomaru Y, Kasukawa T, Waki K, Nakanishi M, Nakamura M, Nishida H, Yap C, Suzuki M, Kawai J (2005). Antisense transcription in the mammalian transcriptome. Science.

[53] Wang H, Chung PJ, Liu J, Jang I-C, Kean MJ, Xu J, Chua N-H (2014). Genome-wide identification of long noncoding natural antisense transcripts and their responses to light in Arabidopsis. Genome Res.

[54] Shen Y, Wang X, Wu F, Du S, Cao Z, Shang Y, Wang X, Peng C, Yu X, Zhu S (2006). The mg-chelatase H subunit is an abscisic acid receptor. Nature.

[55] Kim YC, Nakajima M, Nakayama A, Yamaguchi I (2005). Contribution of gibberellins to the formation of Arabidopsis seed coat through starch degradation. Plant Cell Physiol.

[56] Berthet S, Demont-Caulet N, Pollet B, Bidzinski P, Cezard L, Le Bris P, Borrega N, Herve J, Blondet E, Balzergue S (2011). Disruption of LACCASE4 and 17 results in tissue-specific alterations to lignification of Arabidopsis thaliana stems. Plant Cell.

[57] Alabadi D, Gallego-Bartolome J, Orlando L, Garcia-Carcel L, Rubio V, Martinez C, Frigerio M, Iglesias-Pedraz JM, Espinosa A, Deng XW (2008). Gibberellins modulate light signaling pathways to prevent Arabidopsis seedling de-etiolation in darkness. Plant J.

[58] Oh E, Yamaguchi S, Hu J, Yusuke J, Jung B, Paik I, Lee HS, Sun TP, Kamiya Y, Choi G (2007). PIL5, a phytochrome-interacting bHLH protein, regulates gibberellin responsiveness by binding directly to the GAI and RGA promoters in Arabidopsis seeds. Plant Cell.

[59] Kim DH, Yamaguchi S, Lim S, Oh E, Park J, Hanada A, Kamiya Y, Choi G (2008). SOMNUS, a CCCH-type zinc finger protein in Arabidopsis, negatively regulates light-dependent seed germination downstream of PIL5. Plant Cell.

[60] Lau OS, Deng XW (2010). Plant hormone signaling lightens up: integrators of light and hormones. Curr Opin Plant Biol.

[61] Zhang H, Lin C, Gu L (2017). Light regulation of alternative pre-mRNA splicing in plants. Photochem Photobiol.

[62] Gururani MA, Mohanta TK, Bae H (2015). Current understanding of the interplay between Phytohormones and photosynthesis under environmental stress. Int J Mol Sci.

[63] Stavang JA, Pettersen RI, Wendell M, Solhaug KA, Junttila O, Moe R, Olsen JE (2010). Thermoperiodic growth control by gibberellin does not involve changes in photosynthetic or respiratory capacities in pea. J Exp Bot.

[64] Yuan L, Xu DQ (2001). Stimulation effect of gibberellic acid short-term treatment on leaf photosynthesis related to the increase in rubisco content in broad bean and soybean. Photosynth Res.

[65] Tian J, Song Y, Du Q, Yang X, Ci D, Chen J, Xie J, Li B, Zhang D (2016). Population genomic analysis of gibberellin-responsive long non-coding RNAs in Populus. J Exp Bot.

[66] Dijkstra P, Reegen H, Kuiper PJ (1990). Relation between relative growth rate, endogenous gibberellins, and the response to applied gibberellic acid for Plantago major. Physiol Plant.

[67] Cramer M, Nagel O, Lips S, Lambers H (1995). Reduction, assimilation and transport of N in normal and gibberellin-deficient tomato plants. Physiol Plant.

[68] Xie J, Tian J, Du Q, Chen J, Li Y, Yang X, Li B, Zhang D (2016). Association genetics and transcriptome analysis reveal a gibberellin-responsive pathway involved in regulating photosynthesis. J Exp Bot.

[69] Wieland WF, Wample RL (1985). Effects of paclobutrazol on growth, photosynthesis and carbohydrate content of ‘dDlicious’ apples. Sci Hortic.

[70] Berova M, Zlatev Z, Stoeva N (2002). Effect of paclobutrazol on wheat seedlings under low temperature stress. Bulg J Plant Physiol.

[71] Vu JC, Yelenosky G (1992). Growth and photosynthesis of sweet orange plants treated with paclobutrazol. J Plant Growth Regul.

[72] Jaleel CA, Manivannan P, Sankar B, Kishorekumar A, Sankari S, Panneerselvam R (2007). Paclobutrazol enhances photosynthesis and ajmalicine production in Catharanthus roseus. Process Biochem.

[73] Huang JH, Qi YP, Wen SX, Guo P, Chen XM, Chen LS (2016). Illumina microRNA profiles reveal the involvement of miR397a in Citrus adaptation to long-term boron toxicity via modulating secondary cell-wall biosynthesis. Sci Rep.

[74] Zhou J, Lee C, Zhong R, Ye ZH (2009). MYB58 and MYB63 are transcriptional activators of the lignin biosynthetic pathway during secondary cell wall formation in Arabidopsis. Plant Cell.

[75] Abdel-Ghany SE, Pilon M (2008). MicroRNA-mediated systemic down-regulation of copper protein expression in response to low copper availability in Arabidopsis. J Biol Chem.

[76] Wang CY, Zhang SC, Yu Y, Luo YC, Liu Q, Ju CL, Zhang YC, Qu LH, Lucas WJ, Wang XJ (2014). MiR397b regulates both lignin content and seed number in Arabidopsis via modulating a laccase involved in lignin biosynthesis. Plant Biotechnol J.

[77] Wang ZJ, Ji HT, Yuan BJ, Wang SF, Su C, Yao B, Zhao HT, Li X. ABA signalling is fine-tuned by antagonistic HAB1 variants. Nat Commun. 2015;6:8138–50. 10.1038/ncomms9138 26419884

[78] Borsani O, Zhu J, Verslues PE, Sunkar R, Zhu JK (2005). Endogenous siRNAs derived from a pair of natural cis-antisense transcripts regulate salt tolerance in Arabidopsis. Cell.

[79] Tufarelli C, Stanley JAS, Garrick D, Sharpe JA, Ayyub H, Wood WG, Higgs DR (2003). Transcription of antisense RNA leading to gene silencing and methylation as a novel cause of human genetic disease. Nat Genet.

[80] Swiezewski S, Liu F, Magusin A, Dean C (2009). Cold-induced silencing by long antisense transcripts of an Arabidopsis Polycomb target. Nature.

[81] Arnon DI (1949). Copper enzymes in isolated chloroplasts. Polyphenoloxidase in Beta vulgaris. Plant Physiol.

[82] Moreira-Vilar FC, de Cássia Siqueira-Soares R, Finger-Teixeira A, de Oliveira DM, Ferro AP, da Rocha GJ, Maria de Lourdes LF, dos Santos WD, Ferrarese-Filho O (2014). The acetyl bromide method is faster, simpler and presents best recovery of lignin in different herbaceous tissues than klason and thioglycolic acid methods. PLoS One.

[83] Trapnell C, Pachter L, Salzberg SL (2009). TopHat: discovering splice junctions with RNA-Seq. Bioinformatics.

[84] Trapnell C, Williams BA, Pertea G, Mortazavi A, Kwan G, van Baren MJ, Salzberg SL, Wold BJ, Pachter L (2010). Transcript assembly and quantification by RNA-Seq reveals unannotated transcripts and isoform switching during cell differentiation. Nat Biotechnol.

[85] Wang L, Feng Z, Wang X, Wang X, Zhang X (2010). DEGseq: an R package for identifying differentially expressed genes from RNA-seq data. Bioinformatics.

[86] Merkin J, Russell C, Chen P, Burge CB (2012). Evolutionary dynamics of gene and isoform regulation in mammalian tissues. Science.

[87] Barbosa-Morais NL, Irimia M, Pan Q, Xiong HY, Gueroussov S, Lee LJ, Slobodeniuc V, Kutter C, Watt S, Colak R (2012). The evolutionary landscape of alternative splicing in vertebrate species. Science.

[88] Trapnell C, Roberts A, Goff L, Pertea G, Kim D, Kelley DR, Pimentel H, Salzberg SL, Rinn JL, Pachter L (2012). Differential gene and transcript expression analysis of RNA-seq experiments with TopHat and cufflinks. Nat Protoc.

[89] Shen S, Park JW, Lu ZX, Lin L, Henry MD, Wu YN, Zhou Q, Xing Y (2014). rMATS: robust and flexible detection of differential alternative splicing from replicate RNA-Seq data. Proc Natl Acad Sci U S A.

[90] Conesa A, Götz S, García-Gómez JM, Terol J, Talón M, Robles M (2005). Blast2GO: a universal tool for annotation, visualization and analysis in functional genomics research. Bioinformatics.

[91] Gao Y, Wang H, Zhang H, Wang Y, Chen J, Gu L (2017). PRAPI: post-transcriptional regulation analysis pipeline for Iso-Seq. Bioinformatics.

[92] Sun L, Luo H, Bu D, Zhao G, Yu K, Zhang C, Liu Y, Chen R, Zhao Y (2013). Utilizing sequence intrinsic composition to classify protein-coding and long non-coding transcripts. Nucleic Acids Res.

[93] Ostlund G, Schmitt T, Forslund K, Kostler T, Messina DN, Roopra S, Frings O, Sonnhammer EL (2010). InParanoid 7: new algorithms and tools for eukaryotic orthology analysis. Nucleic Acids Res.

[94] Breton G, Kay SA, Pruneda-Paz JL (2016). Identification of Arabidopsis transcriptional regulators by yeast one-hybrid screens using a transcription factor ORFeome. Methods Mol Biol.

[95] Fan C, Ma J, Guo Q, Li X, Wang H, Lu M (2013). Selection of reference genes for quantitative real-time PCR in bamboo (Phyllostachys edulis). PLoS One.

